# Handball Training and Competition With Facemasks in Galicia: The FISICOVID-DXTGALEGO Protocols Experience

**DOI:** 10.3389/fpsyg.2022.851732

**Published:** 2022-04-07

**Authors:** Javier Rico-Díaz, Dan Río-Rodríguez, Joaquín Gómez-Varela, Rafael Martín-Acero

**Affiliations:** ^1^Facultade de Ciencias da Educación, Universidade de Santiago de Compostela, Santiago de Compostela, Spain; ^2^Grupo de Aprendizaje y Control del Movimiento Humano, Facultade de Ciencias do Deporte e a Educación Física, Universidade da Coruña, A Coruña, Spain; ^3^ATP Entrenamiento Personal, A Coruña, Spain

**Keywords:** infectious disease transmission, facemask, public sport policies, perception, COVID-19

## Abstract

**Objective:**

COVID-19 caused a complete stop in non-professional sports. The use of face masks for team sports is not a widely used measure in non-professional sports. The study aimed to evaluate the perception about using the mask and the adaptation difficulties related to training and competition in team sports following the FISICOVID-DXTGALEGO protocol.

**Methods:**

Seven hundred eighty-seven handball players from the Galician Handball Federation were followed during their return to participation after months of confinement through an electronic questionnaire of perception and experience on the use of a mask.

**Results:**

There is an excellent adaptation to the mask in training and competition with medium and high correlations. The 86,41% of players reported an adaptation to the mask in 3 weeks with a three times a week training frequency. The negative opinion on the mask was drastically reduced (-66.86%) after use. The 80,44% of players considered the use of a mask as an essential measure to resume competitions.

**Conclusions:**

It is very feasible to adapt to training and compete with a mask (hygienic or surgical) in a short period. The use of a mask following these protocols changed previous opinions on the disadvantages of the mask during training and competition.

## Introduction

The practice of amateur sports has been severely affected during the lockdowns due to COVID-19 despite its well-recognized health benefits (Bull et al., 2020; Driggin et al., 2020; Comisión Europea, 2021). Maintaining sports practice during the COVID-19 outbreak is essential due to its effects on the immune system and physical condition, increasing the immune response capacity against the disease (Barker-Davies et al., 2020; da Silveira et al., 2021). However, control over the risk of contagion must be strict (Barker-Davies et al., 2020). Professional sports have returned with high safety investments (DiFiori et al., 2021; Meyer et al., 2021), while amateur and recreational sports suffer from restrictions that will weigh them down for a long time (Timpka, 2020; Ugbolue et al., 2020). The challenge now is to have specific measures for this group, especially considering young people (OECD, 2020) and women (Pons et al., 2020).

Research and opinion polls reported that most of the population is willing to use the mask (Angus Reid Institute, 2020; Tendencias COFARES, 2020; Yermal, 2020; Taylor and Asmundson, 2021); however, it has not been applied as a widespread solution in sports. In Galicia, the challenge has been met through the FISICOVID-DXTGALEGO protocols (Lete Lasa et al., 2021a,b), from deconfinement to the present. These protocols have established COVID-specific operational measures for all sports federations under the command of the Galician Administration (i.e., mandatory use of mask). Its objective was to create specific measures for each sport through a risk analysis methodology with the participation of all stakeholders, which increases the perception of the risk-security continuum (Stokes et al., 2020; Kemp et al., 2021; Mora-Rodríguez and Melero-López, 2021) and its successful implementation (Prasetyo et al., 2020).

Minimizing the risks (Schweizer and Renn, 2019; United Nations, 2019) was critical even in team sports like handball or soccer, with “brief and sporadic contact” (Randers et al., 2021). Handball is characterized by high intermittent efforts (Karcher and Buchheit, 2014; Wagner et al., 2014), with high demands on the respiratory (Romer and Polkey, 2008; Janssens et al., 2013) system causing fatigue (Wells and Norris, 2009), and therefore, an adaptation period must be considered when practicing with a mask (Santos-Silva et al., 2020).

The use of a mask reduces the probability of contagion and does not cause CO_2_ poisoning or O_2_ deficiency (Clapp et al., 2020; Steinbrook, 2020; WHO, 2020a). Experts and entities recommend evaluating and determining the use of a mask while training and competing (Kampert et al., 2020; NCAA Sport Science Institute, 2020; SEMED CGCOM, 2020a,b; American Academy of Pediatrics, n.d.; U.S. Centers for Disease Control and Prevention, n.d.), which has been associated with a lower incidence of COVID-19 (Stutt et al., 2020; Watson et al., 2021). An inverse relationship between protection and breathability along the fascemask spectrum (from cloth to N95) (Hamuy Blanco and Janse van Rensburg, 2020; Janse Van Rensburg et al., 2020) that causes discomfort (Scheid et al., 2020). The available data reported a non-relevant clinical impact on health such as cardiorespiratory, physiological (Fikenzer et al., 2020; Shaw et al., 2020; Epstein et al., 2021; Hopkins et al., 2021; Samannan et al., 2021), perceptual (Hopkins et al., 2021), or cognitive (Spang and Pieper, 2020) parameters. The respiratory rate and the activity of the muscles involved are increased (Lavin et al., 2015; Porcari et al., 2016; Chan et al., 2020), CO_2_ fluctuations alter the respiratory drive (Patel et al., 2022), and although the CO_2_ trapped between the mask and the mouth/nose does not become toxic (Xu et al., 2015), it can trigger an alarm without an actual decrease in available O_2_ (Guyenet and Bayliss, 2015). In some people, this available oxygen variation causes anxiety due to hypersensitivity to the increase in CO_2_ (Smoller et al., 2014). Thus, the mask for a team sport should leave enough space between it and the mouth/nose (Xu et al., 2015) to fit the face well (Alcamí et al., 2020). It should be either elastic or semi-elastic, without it entering the mouth when breathing while avoiding exposing the nose or chin (Martín Acero et al., 2020). An authorized and certified mask must be employed (Ministerion De Consumo, 2020). The choice of the mask implies that the tolerance of each person is assessed before the normal use of the mask (WHO, 2020b), in addition to adjusting the load at the beginning of use:

- Decreasing the time and/or intensity, and/or increasing the pauses (Johnson, 2016).
- Wait for an adaptation after several weeks (Johnson, 2016).
- Athletes with cardiovascular and/or respiratory health conditions should consult their doctor (Scheid et al., 2020; Epstein et al., 2021).

The coping strategy with stressful events such as COVID-19 is individual due to perceived ability, experiences, and sociocognitive biases (Cheng and Tang, 2004). The last published review of 348 studies in 65 journals of 85 countries on the topic of Non-Pharmaceutical Interventions against COVID-19 reported the inexistence of studies on the adaptation of masks during the practice of a team sport (Perra, 2021). Therefore, this research has been proposed with these main objectives: To evaluate the perception of the use of mask and adaptation difficulties in handball training and competition; to know the influence of perceptual responses regarding gender, age, and sporting experience; and finally, to evaluate the change in perception between the time of training without a mask and after using it. The results of this study will serve to objectively inform the new decision making in the control of contagion risks using a mask without the need to entirely suspend the practice of team sports.

## Methodology

This study is carried out within the FISICOVID-DXTGALEGO methodological process (Lete Lasa et al., 2021a) of the Galician Sports Administration (Secretaría Xeral para o Deporte, Xunta de Galicia) with the Handball Galician Federation, implementing together a COVID-19 specific protocol that identifies the possible routes of contagion in sports situations.

### Participants and Recruitment

The whole population of handball players with a federative handball license (4,570 players) was invited for answering the questionnaire. The inclusion criteria were being active players between 9 and 36 years old. At these ages, they regularly competed in Galician handball when the study takes place. A total of 787 active players answered the questionnaire (SE95% = 3.18%), 40.8% women and 59.2% men with a mean age of 17.2 (SD ± 5.2), of which 62.5% were under 18 years of age, and 37.5% were 18 or over.

### Electronic Survey Instrument

The present study examined athletes' self-reported perceptions about using the mask in training and competition contexts. The Galician Sports Administration developed an electronic questionnaire with a group of experts from the university (professor of statistics and research methodology) and health system (Doctors of the General Directorate of Public Health). The aim was to know athletes' assessment and opinions about the use of masks incorporated into their training routines and competition events and their relationship with health in general. It was pilot tested with 80 handball players not included in this analysis. The time required to complete the questionnaire was 5 min. It was sent to the entire population of Galician handball federated players 12 weeks after starting the sport with masks. After that period, the questionnaire was administered from 31/11/20 to 05/12/20, following the CHERRIES recommendations (Eysenbach, 2004).

The questionnaire consisted of 34 questions divided into five sections for the collection of: sociodemographic and player experience data, frequency of training and sports level (Table 1), the impact of the pandemic on the continuity of sports practice, data related to their health that could be related to the use of the mask and finally a section for training and competition on aspects of adaptation to the use of the mask, comfort and perceived effort (Tables 2, 3).

**Table 1:** Participants characteristics.

		**Sex**				**Seniority in sport**										**Self reported respiratory disease**				**Regular smoker?**			
		**Female**		**Male**		**Less 6 months**		**6 months−1 year**		**1–3 years**		**4–6 years**		**More than 6 years**		**No**		**Yes**		**No**		**Yes**	
		***n***	**%**	***n***	**%**	***n***	**%**	***n***	**%**	***n***	**%**	***n***	**%**	***n***	**%**	***n***	**%**	***n***	**%**	***n***	**%**	***n***	**%**
AGE Groups	Under 18	203	63.24	289	62.02	12	57.14	7	50.00	41	87.23	125	89.29	307	54.34	436	63.19	56	57.73	486	66.76	6	10.17
	+18 years	118	36.76	177	37.98	9	42.86	7	50.00	6	12.77	15	10.71	258	45.66	254	36.81	41	42.27	242	33.24	53	89.83
	Total	321		466		21		14		47		140		565		690		97		728		59	

**Table 2:** Perception of the mask in handball training.

**Do you know that your club/federation is applying a protocol, approved by the Xunta de Galicia, of safety measures against the contagion of COVID-19 in your sports practice?**	***n***	**%**
No	7	0.89
Yes	779	99.11
**In reference to the use of the mask in your training, indicate the option that best suits your case**	***n***	**%**
I don't train with a mask	1	0.13
I train with a mask depending on the context	23	2.92
I always train with a mask	763	96.95
**What type of mask do you use to COMPETE?**	***n***	**%**
Medical mask	203	25.86
Fabric mask (washable)	45	5.73
Elastic hygienic mask (washable)	98	12.48
Semi-elastic hygienic mask (keep its shape in front of the face—washable)	429	54.65
FFP2/KN95 mask	10	1.27
**How many times a week do you train with mask?**	***n***	**%**
Once a week	14	1.78
2 times a week	186	23.66
3 times a week	387	49.24
4 times a week	170	21.63
5 times a week	24	3.05
More than 6 times a week	5	64
**How long does your workout last on average?**	***n***	**%**
Between 30 and 60 min	51	6,49
Between 60 and 90 min	534	67.94
More than 90 min	201	25.57
**In your club, team or sports facility: are there rest breaks to breathe without a mask? (keeping the distance of more than 1.5 m)**	***n***	**%**
No	190	24.17
Yes	596	75.83
**Do you consider that you are already adapted to the use of a mask during training?**	***n***	**%**
No	157	21.69
Yes	567	78.31
**How many weeks did it take you to adapt to the use of the mask while training?**	***n***	**%**
1 week	178	31.39
2 weeks	192	33.86
3 weeks	120	21.16
4 weeks	39	6.88
More than 4 weeks	38	6.70
**Before you started wearing the mask in training, WHAT PERCEPTION DID YOU HAVE ABOUT USING IT FOR TRAINING?**	***n***	**%**
1 Very negative opinion	193	24.55
2	226	28.75
3	236	30.03
4	75	9.54
5 Very positive opinion	56	7.12
**CURRENTLY, how do you feel TRAINING with a mask (COMFORT)?**	***n***	**%**
1 Very uncomfortable	73	9.29
2	108	13.74
3	251	31.93
4	268	34.10
5 Very comfortable	86	10.94
**In your training, using the mask do you perceive that your BREATHING (VENTILATION) is negatively altered...?**	***n***	**%**
1 Heavily altered breathing	47	5.98
2	190	24.17
3	230	29.26
4	218	27.74
5 Unchanged breathing	101	12.85
**Using the mask: do you perceive MORE OR LESS EFFORT in TRAINING?**	***n***	**%**
1 Less effort	13	1.7
2	51	6.5
3	225	28.8
4	305	39.0
5 More effort	188	24.0
**Do you think that wearing a mask during training can negatively affect your health?**	***n***	**%**
No	495	68.37
Yes	229	31.63
**How much do you think can negatively affect your health?**	***n***	**%**
1 Nothing at all	4	1.75
2	26	11.35
3	89	38.86
4	76	33.19
5 Very much	34	14.85

**Table 3:** Description of use and perception of the mask in competition.

**Mostly you compete at the level of**		
Regional competitions (only within galicia)	604	90.28
National competitions (in galicia and/or outside galicia)	65	9.72
**Do you wear the mask in competition?**	***n***	**%**
No	18	2.69
Yes	651	97.31
**Do you use a different type of mask training compared to competing?**	***n***	**%**
Different mask	729	92.63
Same mask	58	7.37
**What type of mask do you use to COMPETE?**	***n***	**%**
Basic surgical type mask	4	0.61
Hygienic cloth mask (washable)	8	1.23
Elastic hygienic mask (washable)	73	11.21
Semi-elastic hygienic mask (keep its shape in front of the face—washable)	565	86.79
FFP2/KN95 mask	1	0.15
**CURRENTLY, how do you feel COMPETING with a mask (COMFORT)?**	***n***	**%**
1 Very uncomfortable	68	10.45
2	119	18.28
3	236	36.25
4	194	29.80
5 Very comfortable	34	5.22
**In your competition, using the mask do you perceive that your BREATHING (VENTILATION) is negatively altered...?**	***n***	**%**
1 Heavily altered breathing	24	3.69
2	125	19.20
3	227	34.87
4	189	29.03
5 Unchanged breathing	86	13.21
**Using the mask: do you perceive MORE OR LESS EFFORT in COMPETITION?**	***n***	**%**
1 Less effort	5	0.77
2	37	5.73
3	182	28.17
4	277	42.88
5 More effort	145	22.45
**Since you used the mask IN THE COMPETITION. has your opinion about using it in THESE ACTIVITIES changed?**	***n***	**%**
A lot worse	29	4.45
Much worse	86	13.21
It did not vary	277	42.55
Much better	250	38.40
A lot better	9	1.38
**How important do you think the mask was to resume competition and continue competing?**	***n***	**%**
1 Not important	26	3.56
2	21	2.87
3	96	13.13
4	138	18.88
5 Very important	450	61.56


Closed questions with various response categories were mainly used, except when the numerical response was requested for age. In the case of questions related to perception, a Likert-type scale of five levels were used, with 1 being the lowest value and 5 the highest.

### Data Analysis

All data analyses were conducted using the Statistical Package for Social Sciences (SPSS 26.0, IBM) with a significance level set at *P* >0.05. Data were expressed as mean ±SD, or percentages, as appropriate; percentages were used to compare groups. Another non-parametric measure used to explore association in categorical and ordinal variables was Chi-Square performed to analyze gender groups, age groups, and years of practice related to training context variables and competition, mask use, and perception. Kendall's tau-b was used for ordered values.

### Ethical Considerations

Deputy Director General of Plans and Programs of the General Secretariat of Sports of the Xunta de Galicia approved all the procedures. Data that would allow personal identification was not requested. The athletes who responded to the questionnaire gave their express written consent; in the case of minors, those who had their guardianship were given the written consent. All participants and managers were informed that the data would be used to improve the work carried out in the face of the COVID-19 pandemic by the Administration regarding the health of athletes and citizens in general in compliance with the seventeenth additional provision of Organic Law 3/2018, of December 5, on Protection of Personal Data and guarantee of digital rights.

## Results

### Description of Participants

#### Sports Identification Data

93.77% of the athletes who responded to the questionnaire only practice handball. 71.79% of the athletes surveyed have been practicing the sport for more than 6 years (see Table 1), 85.39% of the athletes stopped doing physical exercise during the period between the declaration of the state of alarm, and 92.28% returned to participation in sport.

#### Description of Use and Perception of the Mask in Handball Training

Considering the distribution by gender, significant differences were found between women and men considering the training variables. The percentage distribution of responses for all questions can be found in Table 2. Regarding the type of mask used, χ^2^ (4, *N* = 785) = 19.913, *p* ≤ 0.01, 34.06% of the women used the medical mask compared to 20.22% of the men, and 58.71% of the men used the semi-elastic hygienic mask, compared to 48.75% of the women. The number of sessions per week, χ^2^ (5, *N* = 786) = 23,732, *p* ≤ 0.0.001, were 3 in 50.78% of men and 48.75% of women; the mean duration of the sessions, χ^2^ (2, *N* = 786) = 6.319, *p* ≤ 05, was 60 and 90 min in 72% of women compared to 64.95% of men. Considering the perception of the use of the mask in training, with respect to the perceived global effort, χ^2^ (4, *N* = 782) = 12.188, *p* ≤ 0.05, 66.09% of the men valued that it was more, or something more than effort compared to 58.62% of women.

Considering the distribution by age in the two established categories of under 18 years (−18) or 18 years and older (+18) in the context of training, significant differences were found between the athletes of (+18) and those of (−18) in relation to the type of mask used, χ^2^ (4, *N* = 785) = 19.391, *p* = 0.001, 64.16% of (+18) used the semi-elastic hygienic mask compared to 48.89% of the (−18 the number of sessions per week who trained with a mask, χ^2^ (5, *N* = 786) = 26.069, *p* ≤ 0.001, were 3 in 54.08% of (+18) vs. 46, 34% of (−18); mean duration of the training sessions with a mask, χ^2^ (2, *N* = 786) = 18.205, *p* ≤ 0.001, between 60 and 90 min, 70.07% of the athletes of (+18) trained compared to 66.67% (−18), considering the perception of adaptation to the mask, χ^2^ (1, *N* = 724) = 14.020, *p* ≤ 0.001, the percentage of (−18) (82.65%) was >70.72% of those of (+18). Regarding the number of weeks required to adapt to the use of the mask, χ^2^ (1, *N* = 567) = 10.880, *p* ≤ 0.05, the (+18) presented higher percentages than the (−18) in 2, 3 and 4 weeks in the perception prior to starting training with a mask, χ^2^ (4, *N* = 786) = 18.398, *p* = 0.001, the percentages of (+18) were higher in negative evaluations, and much compared to (−18) that were higher than (+18) in the intermediate evaluations and on the perception of whether the use of the mask while training negatively affects their health, χ^2^ (1, *N* = 724) = 6.905, *p* ≤ 0.05, 71.80% of the group of (−18) considered that it does not affect them negatively, compared to 62.36% of the group (+18).

The distribution of the responses for the of use and perception of the mask in competition can be found in Table 3. A Kendal's tau-b correlation was run to determine the relationship between participants' previous mask perception for competition, and competition participants comfort wearing mask perception, harmful disturbance of breathing, and general perception of effort during training using a mask. Positive, weak associations were found between previous mask perception for sports practice (training and competition) and participant mask comfort perception in competition context (τ_*b*_ = 0.256, *p* ≤ 0.001), which was statistically significant. Negative, weak associations were found between previous mask perception for sports practice (training and competition) and harmful disturbance of breathing (τ_*b*_ = −182, *p* ≤ 0.001), and general perception of effort during training (τ_*b*_ = −155, *p* ≤ 0.001), both statistically significant. A double-entry table comparing previous users mask perception with the current perception of adaptation can be found in Table 4 shows that despite the previous perception, the current majority situation is to be adapted to the use of mask in competition.

**Table 4:** Double-entry table comparing previous users mask perception with the current perception of adaptation.

**Before you started wearing the mask in training, WHAT PERCEPTION DID YOU HAVE ABOUT USING IT DURING TRAINING?**											
	**1**		**2**		**3**		**4**		**5**		**χ^2^**	**p**
	**n**	%	***n***	%	***n***	%	***n***	%	***n***	%		
**Do you think that you are already adapted to its use?**
No	67	36.4	46	22.7	31	14.3	5	7.0	8	16.3	40.404	0.000
Yes	117	63.6	157	77.3	186	85.7	66	93.0	41	83.7		

Medium and high correlations were found between the same variables in training and competition situations for the use of the mask: Comfort (Kendall's Tau-b = 0.588, *p* < 0.0001) Breathing (Kendall's Tau-b = 0.626, *P* < 0.0001), Effort (Kendall's Tau-b = 0.672, *p* < 0.0001) and Change of Opinion after use (Kendall's Tau-b = 0.838, *p* < 0.0001).

## Discussion

This is the first study on a sport where the mask is mandatory for all practitioners. The study aimed to evaluate the perception of masks and adaptation difficulties in handball training and competition. The main results show an excellent adaptation to the mask in training and competition with medium and high correlations. The vast majority adapted to the mask in 3 weeks with training frequencies of 3 times a week.

### Perception and Time Until the Mask Adaptation in Sport

Wearing a mask while doing sports produces discomfort. Although it has a specific effect on ventilation, effort and perception, our results showed a more significant increase (39.8%) in positive than negative opinion (17.7%) after continuous use in training and Handball competitions of 12 weeks on average. These results are very positive even though handball is trained and competed indoors, which significantly increased perceived discomfort (Liu et al., 2020).

Our results on adaptation are similar between female and male athletes in training and competition. Most athletes were able to adapt to the mask's sporting use in 3 weeks with an average of 3 workouts per week. These results are in line with other studies (Santos-Silva et al., 2020), for example, when the differences when performing an aerobic effort in a group were analyzed of athletes with a restrictive mask (EG) and a control group (CG) without a mask, reaching the EG to equal the CG workload after 3 weeks with cardiorespiratory (Porcari et al., 2016) adaptation. This study also reported an increase of 1.5–2 points on a perceived exertion scale (RPE), which is very similar to other studies (Kido et al., 2013) and in line with our results where 60% of athletes both in training and in competition reported an increase in perceived effort.

### Social Environment and Acceptance of the Use of the Mask

More than 80% of the athletes questioned considered the masks as totally necessary for returning to the practice of their sport. Before their return to participation, previous expectations of athletes with more years of experience had a somewhat more negative perception than those with fewer years of practicing that sport. Our results show the importance of knowing athletes' attitudes before using the mask in their practice since a significant association has been found concerning the perception and assessment of their experience after using it, training and competing. Regardless, the perception of 40% of the athletes improved after regular use. The Galician sports administration has worked together with all the stakeholders to ensure the human factor contributed so that the adopted measures could be accepted and effective (Lete Lasa et al., 2021a).

Our results reinforce the need to create an informed and positive environment (Bhatt et al., 2020; Kasting et al., 2020; Stokes et al., 2020), thus avoiding resistance to using a mask due to lack of comfort avoiding the delay in its proper use, as showed athletes' opinions on the necessity of the specific measures. Regarding the use of a mask, the population's opinion is different depending on the context. A positive majority opinion has been identified in Spain (GTM, 2020; Tendencias COFARES, 2020) between 18 and 25 (74.2%). However, negative attitudes toward masks found in the US and Canada formed a network (Taylor and Asmundson, 2021). The central nodes were first to believe that masks were not effective, which is in line with other studies in the US and UK. In these studies, respondents who considered masks as an effective measure did not exceed 40% (Bhatt et al., 2020; Geldsetzer, 2020; Kasting et al., 2020; Samannan et al., 2021). Thus, a node of psycholgical to facemasks reactance was conformed due to mandatory use (Taylor and Asmundson, 2021). In the USA (Pierce et al., 2020), more than 10,000 parents of athletes were surveyed in 45 states, from 13 sports, resulting in perception favoring masks of only 24.3% and against 33%. The sports managers of Galicia significantly improved their ability to identify and perceive the risk of contagion and declared high satisfaction with the participatory methodology FISICOVID-DXTGALEGO (Lete Lasa et al., 2021b). In China, it was also found that cultural differences and people's perception styles include their ability to cope with stressful events related to COVID-19 (Cheng and Tang, 2004), so it is reasonable to think that there are athletes with different styles of coping with stress in the face of stress. COVID-19 (Lete Lasa et al., 2021b), there may also be different response levels to sports mask use.

### Practitioners: Difficult Return to Sports Participation

In Galicia, the pandemic suspended the sporting activity of contact sports for about 6 months, which, together with the uncertainty, was foreseeable that it would cause the participants to either change their sport (Choi and Bum, 2020) or abandon it. The efforts of all socio-sports agents have allowed the maintenance of 55% of Handball practitioners in Galicia. Forty-Nine percentage of those under 18 years of age have returned, a percentage expected by the federative managers of Galicia (Lete-Lasa et al., 2020). Maintaining this number of practitioners during crisis (Brooks et al., 2020) is due to their motivation and because 99% of athletes have and know the FISICOVID_DXTGALEGO protocol. This has highlighted the continuum of risk and safety perceived by families and athletes. Exiting the restrictions caused by COVID-19 is a challenging and essential success that means recovering and stopping the abandonment of the practice of physical-sporting activity, with the associated physical, psychological and emotional benefits associated with it (Hughes, 2020). While in the neighboring country of Portugal, the most popular team sports (handball, basketball, soccer/football, futsal, roller hockey) have retained only the 20.9% of athletes in early ages of sports training (Barbosa, 2020).

## Practical Applications

The following practical applications are extracted from our results:

- The participation of stakeholders in the protocols is necessary for better compliance with the rules.
- It is feasible to adapt to training and competition quickly with a mask.
- Considering the 3 weeks necessary for adaptation to the mask, Starting with shorter periods of effort than regular training and competition would be advisable, reaching a greater number of practitioners with a favorable opinion.

## Limitations

Although athletes were instructed to use multiple masks if the current one was too wet, it wasn't controlled and this might affect the perception of athlete's adaptation to the mask. Also humidity and temperature of the pavilion were not controlled. This issues should be considered in future investigations on this subject.

## Data Availability Statement

The raw data supporting the conclusions of this article will be made available by the authors, without undue reservation.

## Ethics Statement

Ethical review and approval was not required for the study on human participants in accordance with the local legislation and institutional requirements. Written informed consent to participate in this study was provided by the participants or participants' legal guardian/next of kin.

## Author Contributions

JR-D and his collaborators managed data collection. JG-V and RM-A performed data analysis and interpretation. RM-A and DR-R prepared the draft of the article. All authors equally contributed to the design of the study, performed critical revision, and final approval. All authors contributed to the article and approved the submitted version.

## Conflict of Interest

DR-R was employed by company ATP Entrenamiento Personal. The remaining authors declare that the research was conducted in the absence of any commercial or financial relationships that could be construed as a potential conflict of interest.

## Publisher's Note

All claims expressed in this article are solely those of the authors and do not necessarily represent those of their affiliated organizations, or those of the publisher, the editors and the reviewers. Any product that may be evaluated in this article, or claim that may be made by its manufacturer, is not guaranteed or endorsed by the publisher.
